# DeepScratch: Single-cell based topological metrics of scratch wound assays

**DOI:** 10.1016/j.csbj.2020.08.018

**Published:** 2020-08-29

**Authors:** Avelino Javer, Jens Rittscher, Heba Z. Sailem

**Affiliations:** a Institute of Biomedical Engineering, Department of Engineering Science, Old Road Campus Research Building, University of Oxford OX3 7DQ, UK; b Big Data Institute, University of Oxford, Li Ka Shing Centre for Health Information and Discovery, Old Road Campus Research Building, Oxford OX3 7LF, UK

**Keywords:** wound healing, image analysis, migration tissue toplogy, endothelial cells, CDH5, CDC42

## Abstract

Changes in tissue architecture and multicellular organisation contribute to many diseases, including cancer and cardiovascular diseases. Scratch wound assay is a commonly used tool that assesses cells’ migratory ability based on the area of a wound they cover over a certain time. However, analysis of changes in the organisational patterns formed by migrating cells following genetic or pharmacological perturbations are not well explored in these assays, in part because analysing the resulting imaging data is challenging. Here we present DeepScratch, a neural network that accurately detects the cells in scratch assays based on a heterogeneous set of markers. We demonstrate the utility of DeepScratch by analysing images of more than 232,000 lymphatic endothelial cells. In addition, we propose various topological measures of cell connectivity and local cell density (LCD) to characterise tissue remodelling during wound healing. We show that LCD-based metrics allow classification of CDH5 and CDC42 genetic perturbations that are known to affect cell migration through different biological mechanisms. Such differences cannot be captured when considering only the wound area. Taken together, single-cell detection using DeepScratch allows more detailed investigation of the roles of various genetic components in tissue topology and the biological mechanisms underlying their effects on collective cell migration.

## 1 Introduction

Cell migration plays an important role in both tissue repair and disease. For example, increased migratory ability in cancer cells can lead to invasion and metastasis, which are the main causes of cancer mortality [1]. In contrast, regeneration and wound healing require the collective movement of cells to close epithelial gaps [2]. Furthermore, cell migration contributes to sculpting and maintaining tissue architecture and topology by coordinating temporal and spatial cell behaviour. Although molecular factors regulating cell migration at the single-cell level are fairly well understood, research on the molecular factors regulating collective cell motility has been limited by challenges in image analysis [3].

Scratch assays, or wound healing assays, are widely used for assessing collective cell migration. Typically, they are based on scratching the middle of a cell monolayer, inducing collective cell migration toward ‘healing’ or closure of the resultant gap. Multiple automated image analysis methods have been developed for segmenting the wound area [4], [5]. However, these approaches do not provide information on cell-level mechanisms, such as changes in proliferation rate, cell morphology and size, or tissue topology [6]. Consequently, which of these changes are reflected in the closure of a wound following genetic or pharmacological perturbation is often unknown. Single cell analysis can provide more insight into these mechanisms but can be challenging due to limitations on the resolution of images captured in wound healing assays. Therefore, developing single cell analysis methods for scratch assays is critical for gaining more insights into the factors impacting cellular motility.

Advances in deep learning revolutionised our ability to segment and classify cell images, even in challenging datasets [7]. For example, fully convolutional networks learn thousands of features through multiple hierarchical layers to predict the class of individual pixels (e.g. foreground or background) [8]. U-Net is a fully convolutional neural network that is widely used for segmenting biomedical imaging data [9], [10], [11], [12]. Numerous studies have successfully used U-Net architecture to segment nuclear and cellular images, including the Nuclei Segmentation Challenge [13], [14]. CellProfiler 3.0, a widely used toolbox for cell segmentation allows training a U-Net model through the ‘ClassifyPixels’ module [15]. However, segmentation using deep learning approaches requires manually drawing masks for tens to hundreds of cells, which is highly time-consuming. U-Net can also be trained to detect cell locations, rather than classifying every pixel in an image [16], [17]. This strategy only requires dot annotations, which are less time-consuming to obtain and, therefore, are more suited to high-throughput applications, where images are of limited resolution [18].

One aspect of wound closure that can be quantified from single-cell locations in scratch assays is tissue topology. Tissue topology describes the connectivity among cells and is quantified based on the number of neighbours a cell has [19], [20]. It has been intensively investigated in the context of two-dimensional epithelial sheets [21], [22], [23], [24]. In general, epithelial tissues follow consistent topologies; cells tend to adopt a hexagon shape and have six neighbours in both plant and animal epithelia. In addition, the distribution of the number of neighbours, and hence the number of polygon sides, in different types of epithelial tissue tend to be fixed [3]. For example, in the *Drosophila* wing disc the distribution of polygon shapes is approximately 3% ﻿tetragons, 28% pentagons, 46% hexagons and 20% heptagons [25]. Topologies of endothelial cells, a subtype of epithelia that lines the circulatory system, are yet to be determined. Another aspect of tissue topology is local cell density, which affects the distance between neighbours. We and others have shown that local cell density can modulate cell fate via its effect on transcriptional activities [26], [27], and its perturbation is associated with cancer pathways [26], [28]. Surprisingly, how the topology of cell monolayers in scratch assays changes during wound healing is not well explored.

DeepScratch builds on advances in deep learning to detect single cells in scratch wound assays. To our knowledge, DeepScratch is the first network to detect cells from heterogeneous image data using either nuclear or membrane images. Using this approach, we can extract various topological measures from scratch assays, allowing more effective characterisation of cellular mechanisms. To illustrate the utility of DeepScratch, we applied it to a publicly available scratch assay dataset of wild type, and genetically perturbed lymphatic endothelial cells. Specifically, we investigated the effects of CDH5 and CDC42 gene knockdowns that are known to affect endothelial cell migration. However, these two genes act on different biological mechanisms. CDH5 affects cell–cell adhesion, and CDC42 is necessary for protrusion formation in addition to cross-talk with cadherins [29], [30], [31]. Analysis of two-dimensional endothelial layers using DeepScratch revealed that, consistent with their distinct functions, CDC42 and CDH5 affect tissue topologies differently. In summary, we present here a novel pipeline, combining single-cell detection via neural networks with biologically relevant metrics for scratch assays to better characterise cellular mechanisms underlying perturbation effects on collective cell migration.

## 2 Materials and Methods

### 2.1 Dataset

**Fig. 1:**
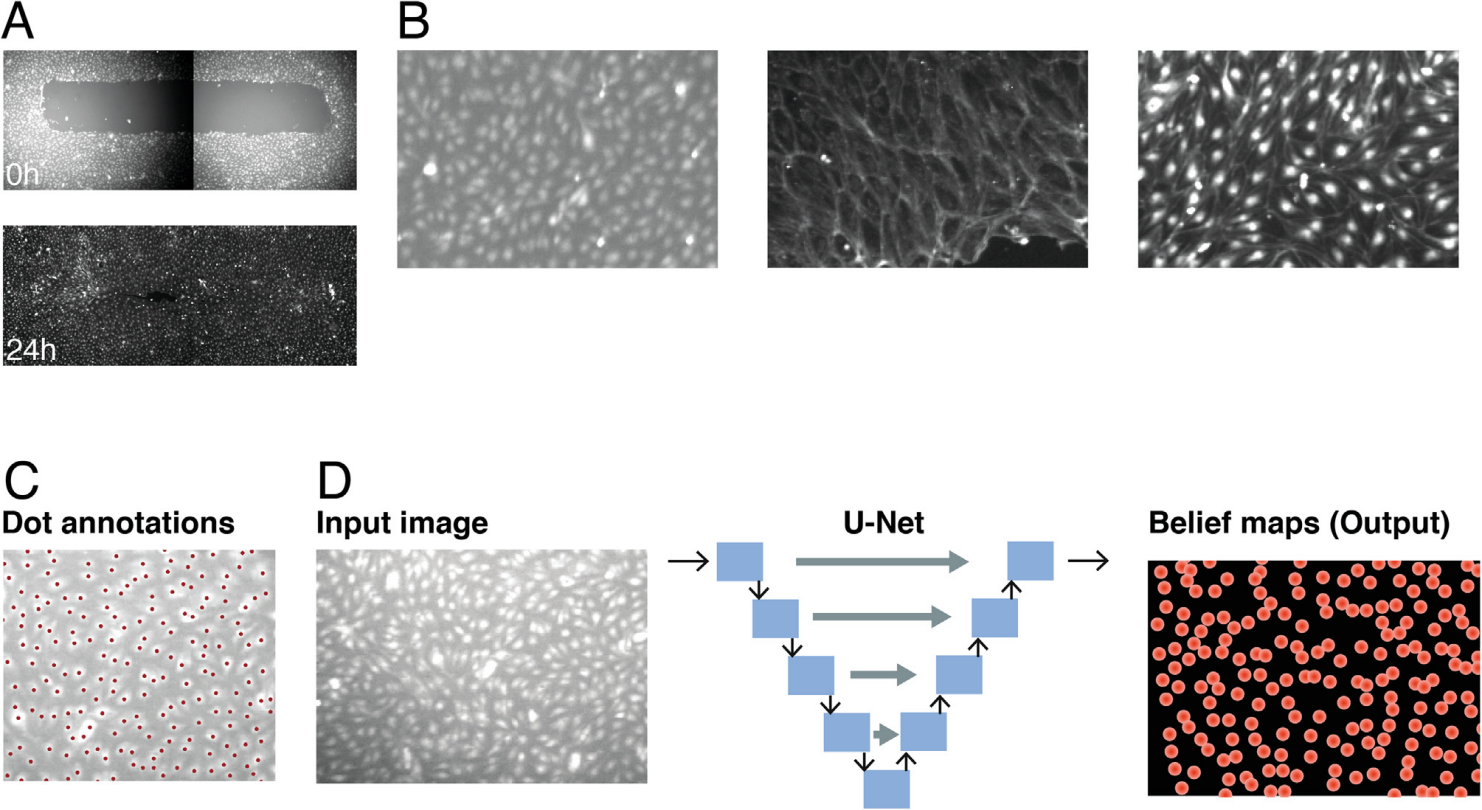
**Training data and DeepScratch model. A)** HDLECs imaged at 0 h and 24 h after scratch-wound assay (4x objective) resulting in two images that were stitched together. **B)** Training data is composed of images in which cells are stained for nuclei, membrane, or both. **C)** Example of dot annotations (red dots). **D)** DeepScratch architecture where each blue rectangle is composed of two convolutional layers, each followed by a leaky ReLU operation and either max-pooling or up-convolution. The input to the network is an image of 96x96 pixels, and the output is a belief map indicating the distance of each pixel to the annotated cell centre. (For interpretation of the references to colour in this figure legend, the reader is referred to the web version of this article.)

Images of human dermal lymphatic endothelial cells (HDLECs) at 0 h and 24 h following a scratch assay were obtained from Williams et al. [30] (Fig. 1A). Cells were stained either for nuclei or membrane or for both (Fig. 1B). The images were acquired at 4x objective, which allowed the entire well to be captured in two images that were stitched together, resulting in 512×1392-pixel images. Three conditions were considered: mock-transfected control cells and knockdown of either CDH5 or CDC42 genes using siRNA. Fifteen technical replicates were analysed per condition, with two timepoints captured (90 images in total). These images were composed of 232,000 cells.

### 2.2 Cell detection using convolutional neural networks

#### Training data

Patches of 192×192 pixels were generated, resulting in 1,740 patches. As manual image annotation was not feasible for all images, only 544 random patches were dot annotated (Fig. 1C). Because cells were stained with different markers, we split the training images into two sets: 1) images with nuclear stain only (‘Nuclei’ set) and 2) images with membrane stain only or both membrane and nuclear stain (‘Mix’ set). In total, 176 patches (22,300 cells) were sampled from the Mix dataset and 368 images (31,440 cells) from the Nuclei dataset. We used 90% of the annotated images for training and split the remaining 10% equally into testing and validation datasets. We used the remaining unannotated data (69% of the data) as an additional testing dataset, for which results were qualitatively assessed. We also assessed the performance on unseen conditions (50 images) and found that DeepScratch was robust to variation in cell shape and intensity variation.

#### Image normalisation

Images were normalised by dividing all pixel values by the maximum possible intensity value (i.e., 2^12^ –1 = 4,095 for 12-bit images).

#### DeepScratch architecture

DeepScratch is based on U-Net architecture [9], which is composed of an autoencoder that downsamples the input image into a compressed representation, followed by a series of upsampled layers that decompress the learned feature maps to the same size as the original image. Skip connections are added to provide contextual information from the feature maps in the downsampling layers to the upsampling layers. As a result, U-Net combines local and global image information to map each pixel to a semantic value. We used four layers for both the downsampling and upsampling branches. In our network, each convolution is activated by a leaky rectified linear unit (ReLU) with a negative slope of 0.1 and no batch normalisation. All convolutions are 3x3 pixels, except for the initial block, where a 7x7 convolution is used. The downstream encoder consists of four blocks, each with two convolutional layers and with 48 feature maps followed by 2x2 max-pooling. The upstream decoder has a similar configuration but replaces max-pooling operations with 2x2 upsampling via nearest neighbour interpolation. Following the final upsampling layer, a final convolution layer compresses the 48 channels into a single channel. We also tested other configurations, including a deeper U-Net (10 and 12 layers) and batch normalisation. We also tested using ResNet architecture [32] in the downsampling branch. These variations did not result in improved performance, which indicates that increasing model complexity does not always increase the accuracy of convolutional networks.

#### DeepScratch training

##### Target objective

To robustly detect cells in the images, we trained U-Net to predict a belief map of the probability that a cell is localised at a given pixel, instead of an absolute location. During training, belief maps are generated by transforming coordinates into pixel-wise annotations and convolving the resulting mask with a Gaussian kernel of size 2.5×2.5 pixels. The size of the kernel is determined empirically and depends on cell size. At inference time, each cell’s coordinates are extracted from the predicted masks by identifying the local maxima. This method is used commonly in pose estimation [16] and has been applied previously to cell localisation [17]. The Gaussian kernel is used to facilitate the network convergence. Additionally, the probability assigned to neighbouring pixels conveys the uncertainty associated with the exact coordinates of the cell centre.

##### Batch construction

Images were randomly selected in each batch. We balanced the number of samples that were seen by the model from the Nuclei and Mix set. A random crop of size 96×96 pixels was selected from each image. The small crop size was appropriate because the global context is not very important for the task, i.e., we do not expect the global shape of the wound to affect the model’s ability to locate an individual cell. Crops were chosen randomly with a 20% probability. The remaining crops were generated by randomly selecting a cell, then choosing a crop including the corresponding cell (80% probability). We used these two approaches for crop selection to ensure that most crops contained cells, while allowing for the possibility that a crop was void of cells.

##### Data augmentation

Data augmentation is a standard practice in training neural networks for making a model more robust to variations that could realistically occur. We augmented each crop by random rotation, horizontal and vertical flipping, or by scaling by a factor between 0.9 and 1.1. Augmentation of the target data is done in the coordinates space, then coordinates are ultimately transformed into belief map space using a Gaussian kernel of size 2.5×2.5 pixels. To account for the high variation in intensity in the dataset, crops were also perturbed by multiplying their intensity values by a random variable between 0.5 and 1.3 or by subtracting a random variable between −0.2 and 0.2.

##### Training parameters

The model was trained using the Adam optimiser with a learning rate = 1.28x10^4^ and batch size = 128 and using minimum squared error as the loss function. The model typically converges after 160 epochs, with each epoch requiring around 3–4 min. After each epoch, the model is saved, and its performance is recorded. We selected the model from the epoch with the lowest error score based on the validation set.

Models were trained on a desktop workstation with NVIDIA TeslaK40c GPU with 12 GB RAM and required approximately 17 h for training. Once trained, the model can process 48 images (512×1392 pixels each) per minute, including postprocessing of the belief maps and segmenting wounds (see below). Thus, this approach is scalable to large datasets.

##### Postprocessing of predicted belief maps

To calculate cell localisation, local maxima are computed from the predicted belief maps. First, predicted belief maps are normalised by the global maxima. Then, max-pooling with 3 × 3 pixel kernel is applied to the resultant image. The intersection between the normalised belief map and its max-pooled version allows the detection of local maxima because only the local maxima are present in these two masks. Finally, only the local maxima above a user-defined threshold are retained. To identify correct predictions, the predicted localisations are matched to the ground truth annotations using the Hungarian method. Then, cells are matched to their nearest neighbour, given that they are within 10 pixels from the annotated points.

##### Wound segmentation

Segmentation of a wound region was done via detection of areas that did not contain cells (Fig. 3A). We first estimated cell density by converting cell density by converting coordinates of cells into a mask with pixel-wise annotations and applying a uniform 13 ×13 pixel kernel. Then, we created a segmentation mask by applying a morphological opening with a 35 × 35 pixel kernel and categorising any black pixel as part of the wound area. We identified all connected components in the wound mask and defined the wound as the object with the largest area.

#### 2.3 Benchmarking

We benchmarked DeepScratch against traditional thresholding methods for cell segmentation using CellProfiler [33]. We used Robust Background thresholding method as it resulted in best segmentation results on our Nuclei set. Detecting cells stained with a heterogeneous set of markers is not feasible using CellProfiler, because a different method needs to be implemented for each marker and, thus, requires a classification step for the marker itself.

### 2.4 Image and data analysis

#### Polygon shapes

The number of sides of each cell was obtained using Voronoi tessellation. This method also reflects the number of neighbouring cells, which allowed approximate segmentation of cells in confluent monolayers. Voronoi tessellation takes as an input the xy coordinates of seed points and assigns every pixel in an image to the nearest seed. Pixels that are equidistant to multiple seeds form the sides of the Voronoi cells. This process results in a diagram filled with adjacent convex polygons (Fig. 3B) and is well-suited to the problem, as epithelial tissues resemble Voronoi organisation.

#### Spatial correlation analysis

To determine whether certain shapes co-occur more often than expected by chance, we estimated the probability of co-occurrence of a polygon with N sides with other polygon shapes, based on a random distribution (Table 1). For example, based on the relative frequency of hexagons in our images, the expected probability that a hexagon is in contact with another hexagon is 36%. Thus, based on a random distribution of polygon shapes, each hexagon is expected to be in contact with approximately two other hexagons, assuming a random distribution (36% of 6 sides = 2.16 sides). Deviation from expected rates of co-occurrence (Table 1) indicates potential clustering.

**Table 1:** Percentages of sides that should be shared between polygon shapes based on a random distribution.

Polygon shape	Tetragon	Pentagon	Hexagon	Heptagon
Tetragon	**0.28**	1.16	2.16	0.8
Pentagon	0.35	**1.5**	1.8	1.0
Hexagon	0.42	1.47	**2.16**	1.2
Heptagon	0.49	2.03	2.52	**1.4**

To compute the actual co-occurrence probability, we determined the frequency of co-occurrence between various polygon shapes. These frequencies were normalised to their sum. Therefore, the probabilities that are shown in Fig. 3E-H were normalised by row.

#### Local cell density (LCD)

Cells that are closer to their nearest neighbours have higher densities and vice versa, so we used distance to the nearest neighbour to measure local cell density. We tested various numbers of nearest neighbours, which generally had similar results (results not shown). The distance to the nearest 36th neighbour was selected to capture both direct and indirect neighbours, and LCD was computed as the inverse of the distance to the nearest 36th neighbour. As LCD varies depending on distance to the wound centre, we defined normalised LCD as the ratio between LCD and distance to the wound centre. Because the wound was completely closed by cells at t_24h_ under some conditions, distance to the wound centre was estimated at t_0h_.

#### Principle component analysis (PCA)

We computed LCD based on distance to the nearest 36th neighbour and 10th neighbour and on the area of Voronoi cells to account for different length scales. To obtain more information on single-cell distribution and heterogeneity within the well, we calculated the median, skewness, standard deviation and 10%, 25%, 75% and 90% quantiles of LCD [28]. We applied PCA to the fold change (t_24h_/t_0h_) of these different statistics.

## 3 Results

### 3.1 Robust detection of cell localisation using DeepScratch

**Fig. 2:**
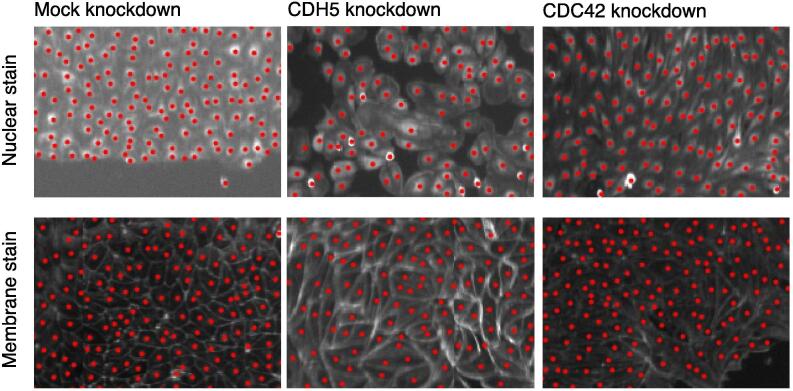
**DeepScratch robustly detects cells across different stains and conditions.** Top row includes images with nuclear stain while bottom row includes images with membrane stain.

We implemented DeepScratch, an optimised U-Net, to localise cells in scratch assays when nuclei or membrane markers are used (Methods and Fig. 1). DeepScratch accurately detected cells in both membrane and nuclei images under different treatment conditions that affected cell shape or adhesion (Fig. 2). We assessed its performance using the F-score metric, which accounts for both sensitivity and specificity. DeepScratch scored 95.8% when considering both Nuclei and Mix set and 98.2% on Nuclei set only (Table 2). As expected, the model score decreased when the set contained membrane images, as detecting cells in some of the images was challenging even for an expert. A more complex model, using a ResNet in the downsampling branch, did not improve results (Methods). Furthermore, the performance of DeepScratch is significantly better than CellProfiler, which uses traditional thresholding techniques (Methods). In particular, thresholding approaches resulted in significantly higher false-negative rates when image contrast was low as reflected by the lower recall (Table 2). These results show that DeepScratch is a robust approach for single cell detection in scratch assays and, unlike traditional pipelines, is generalisable to multiple stains, allowing for increased flexibility in experimental design.

**Fig. 3:**
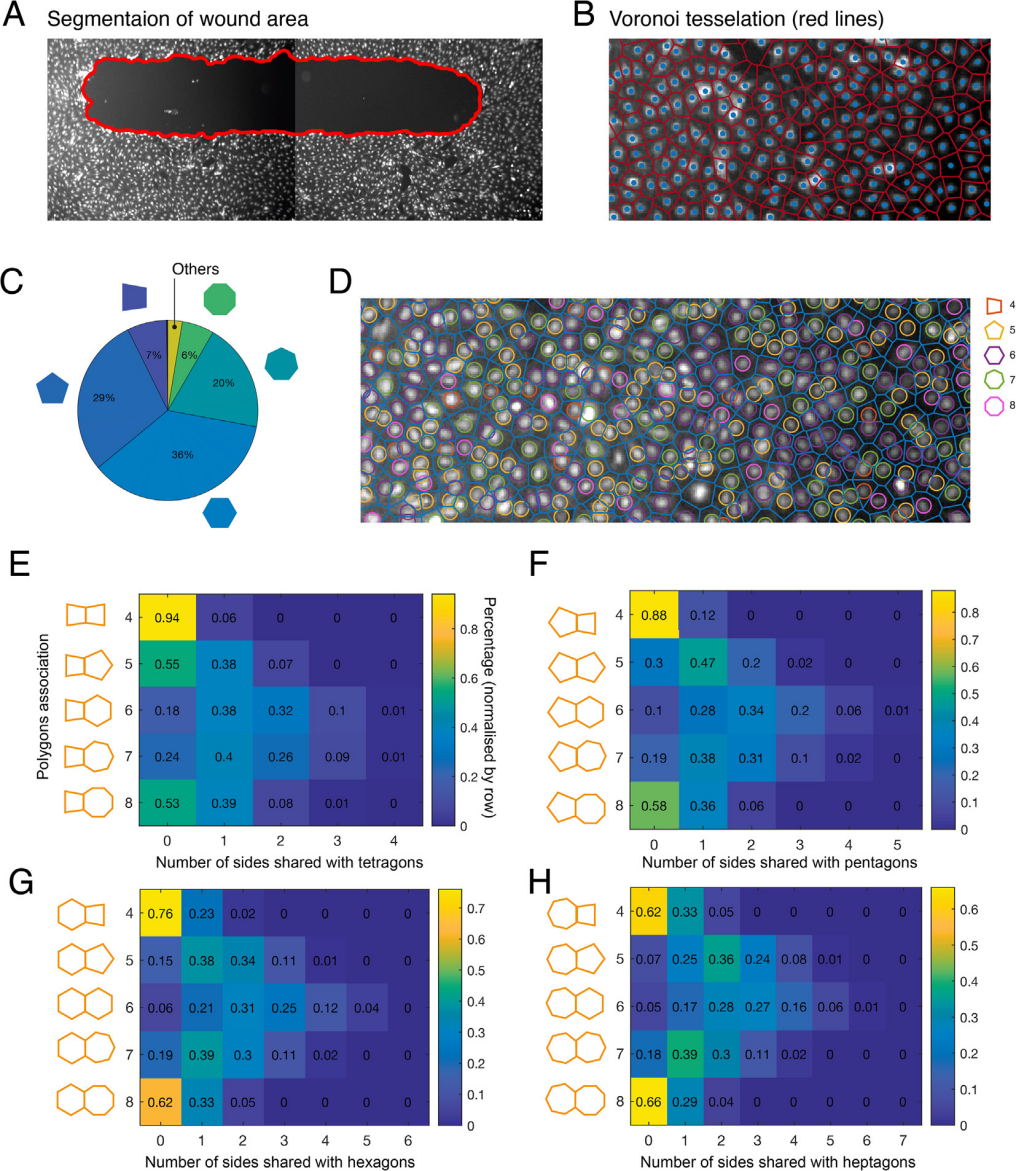
**Endothelial cells topology. A)** Segmentation of wound area **B)** Voronoi diagram reflecting the number of neighbours for each cell and approximate morphology, such as area and elongation. **C)** Percentages of different polygon shapes in endothelial confluent monolayers (t_0h_). **D)** Example on the distribution of different polygon shapes in the well. More hexagons (purple) on the right (64) of the image than the left (38). **E-H)** Heatmaps of co-occurrence probabilities of different polygon shapes with pentagons (E), hexagons (F), heptagons (G) and octagons **(H)** as indicated on the left of each heatmap. Frequencies are normalised by row. (For interpretation of the references to colour in this figure legend, the reader is referred to the web version of this article.)

**Table 2:** DeepScratch performance results.

Image data	Nuclei Set			Mix Set			Nuclei + Mix Set		
	F-score	Precision	Recall	F-score	Precision	Recall	F-score	Precision	Recall
DeepScratch – Simple	98.2%	97.1%	97.4%	92.5%	91.7%	92.1%	95.8%	95.4%	96.2%
DeepScratch – ResNet	96.0%	96.1%	89.3%	92.5%	96.1%	89.3%	94.3%	96.1%	89.3%
CellProfiler	87.84%	96.75%	80.4%	–	–	–	–	–	–

### 3.2 Endothelial cells are constrained topologically

Because cell–cell connectivity can play an important role in collective cell migration, we first investigated whether the topology of endothelial monolayers follows certain patterns. Using the cell locations detected by DeepScratch, we determined the distribution of different polygon shapes, which itself indicates the number of neighbouring cells. At t_0h_, when wound response at its onset, the topology of cells away from the wound should reflect the organisation of cells in steady-state. Polygon shape can be determined based on Voronoi tessellation (Fig. 3B and Methods). On average, each image in our dataset was composed of approximately 3000 cells (+500 at t_24h_), which provided sufficient data for our analyses. Wounds were segmented, and cells at the wound and image edges were excluded from further analysis. We found that the most frequent topology in HDLECs at t_0h_ is a 6-sided polygon (Fig. 3C), similar to what is reported for epithelial tissues [21]. The mean percentage of hexagonal cells is 36.16%. Pentagons are the second most-frequent topology with (mean = 28.64%), followed by heptagons (mean = 19.55%). Tetragons and octagons are detected at similar relative frequencies (6%-7%). Consistent with previous work, larger cells tend to be in contact with more cells and, hence, more sides (Pearson correlation coef. = 0.435, *p*-*value* < 0.00001) [21]. These results suggest that the distribution of different polygon shapes is constrained in HDLECs, and hexagons are the most frequent shape.

We explored whether cells with a similar number of sides or certain topologies tend to cluster together (i.e. are spatially correlated) or to spread randomly in the well. Qualitatively, we observed that certain image regions tended to contain more of a particular shape than neighbouring regions. For example, more 6-sided polygons can be seen in the right side of the image in Fig. 3D than on the left. To identify potential spatial correlations between topologies, we computed the probability of co-occurrence between different shapes (Methods and Fig. 3E-H), where deviation from expected values (Table 1) indicates clustering behaviour. We found that pentagons are most likely to share a single side with other pentagons (47%), while 20% of pentagons shared 2 sides with other pentagons, and 30% did not share any side with another pentagon (Fig. 3E). These results are reasonably consistent with the relative occurrence of pentagons, where a pentagon is expected to share 1.5 sides with another pentagon. Alternatively, pentagons shared 1 or 2 sides with heptagons with similar probabilities of 38% and 31% respectively. This result deviates from expected frequencies, where pentagons are expected to share only a single side with other heptagons, based on their relative frequency. Additionally, hexagons tended to cluster together, sharing 2, 3 or 4 sides with other hexagons with probabilities of 31%, 25% and 12% respectively (Fig. 3G). These results suggest that similar polygon shapes are not extremely clustered, with the exception of hexagons, and that some topologies tend to be more correlated with each other.

### 3.3 Tissue remodelling during wound healing

**Fig. 4:**
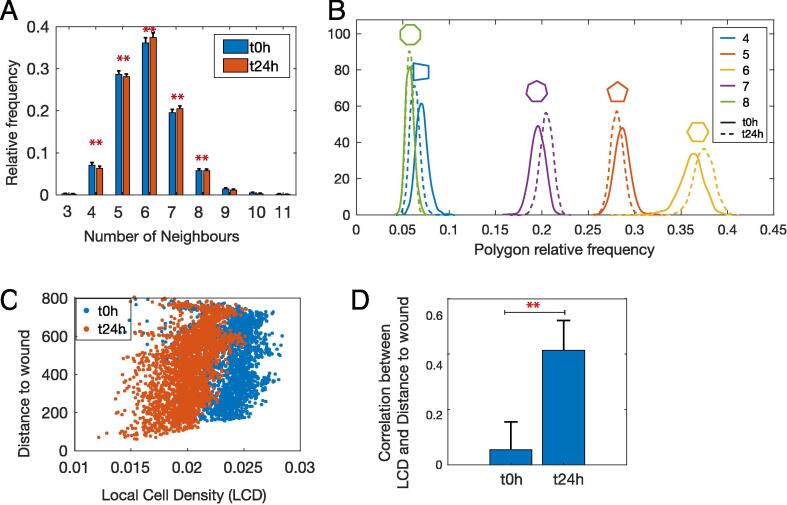
**Remodelling of tissue topology during wound healing. A)** A small but significant change in the average relative frequency of polygon shapes after 24 h of scratch assay (*p*-value < 0.00001). **B)** Changes in the distribution of polygon shapes. Solid and dashed lines indicate distribution at t_0h_ and t_24h_ respectively. **C)** Local cell density (LCD) versus distance to wound centre of single mock-transfected cells from one well at t_0h_ and t_24h_ shows increased LCD during wound healing (n = 1). **D)** Pearson correlation coefficient between LCD and distance to wound centre is significantly higher at t_24h_ (n = 15, ** indicates *p-value* = 8.8e-07).

We determined whether topological metrics can be useful for quantifying changes during wound healing and observed a small but significant change in the distribution of polygon shapes at 24 h following wounding (Fig. 4A*, p-value* < 0.00001). Specifically, hexagons increased by 1.29% and heptagons increased by 0.95%, while the frequencies of other polygons decreased (Fig. 4A-B). These results suggest that cell–cell connectivity based on the distribution of different polygon shapes does not provide a sensitive metric of collective cell migration in scratch assays.

Next, we investigated changes in local cell density (LCD) during wound healing as another measure of cell topology (Methods). LCD indicates the number of cells per unit area and reflects both cell spreading (lower LCD) and proliferation (higher LCD) and can be independent of the number of cells. LCD significantly decreased 24 h following scratch assay, which reflect cell spreading as they migrate to close the created gap (Fig. 4C). LCD was also positively correlated with distance to wound centre (Fig. 4D, Pearson correlation = 0.4). Cells had lower density at the leading edge of the wound and higher density as distance from the centre increased (Fig. 4C). Interestingly, this correlation was not observed at t_0h_. These results show that cell spreading is spatially coordinated during wound healing and suggest that measures of local density can provide a useful metric in scratch assays.

### 3.4 Topological metrics for characterising perturbation effects in scratch assays

**Fig. 5:**
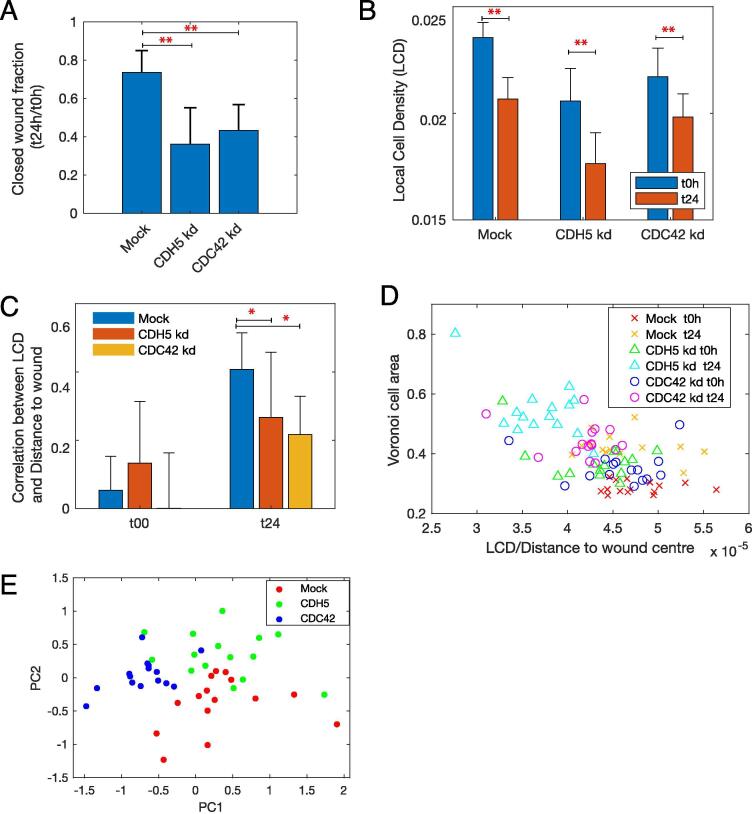
**Local cell density provide biologically relevant metrics of wound healing assays. A)** Fold change of wound area (t_24h_/t_0h_) is significantly lower in CDH5 or CDC42 depleted cells. Kd: knockdown (** indicates *p*-*value* < 4.2e-05). B) LCD is significantly lower at t_24h_ in all conditions but is lowest in CDH5 knocked-down cells (** indicates *p*-*value* < 2.3e-04). **C-D)** Correlation between LCD and distance-to-wound-centre is significantly different between conditions at t_24h_ but not at t_0h_ (* indicates *p-value* < 0.02). **E)** Fold change of LCD-derived metrics (t_24h_/t_0h_) projected into the first two principle components show that depletion of CDH5 and CDC42 have distinct effects on tissue topologies, confirming their distinct biological mechanisms.

We sought to determine whether LCD can distinguish between genetic perturbations affecting different cellular mechanisms using CDH5 and CDC42 knockdowns. Both knockdowns resulted in significant decrease in wound area closure, compared with mock-transfected cells (Fig. 5A). Like mock-transfected control samples, average LCD decreased in CDC42 and CDH5 depleted cells (Fig. 5B). These results indicate that a decrease in cell density is due to cell spreading and is independent of cell migration. Indeed, CDH5 had the lowest LCD as the loss of cell–cell adhesion resulted in larger cells (Fig. 2). However, the correlation between LCD and distance to wound centre was significantly less in CDH5 and CDC42 knockdowns compared to mock samples (Fig. 5C and *p-value* < 0.02). We also observed different behaviour from cells in these different conditions when considering both Voronoi cell area and LCD normalised to distance to wound centre (Fig. 5D). Therefore, we computed different statistics based on normalised LCD (Methods). We visualised the fold change of these measures (t_24h_/t_0h_) in reduced principal component space to identify their variation between conditions. CDH5 and CDC42 knockdowns not only clustered away from mock-transfected cells but also displayed distinct topological signatures (Fig. 5E). These results show that measures derived from single-cell densities can improve characterisation of genetic perturbation effects on cell migration.

## 4 Discussion

Scratch-wound assay is widely used in biomedical studies to assess cell motility. However, analysis of the resultant imaging data has been limited to wound area. We developed DeepScratch to accurately localise cells in scratch assay images, allowing the generation of rich feature sets from these images and investigation of various biological questions. We have shown here that DeepScratch outperforms traditional analysis methods. Importantly, in contrast to current methods, our approach is highly flexible and can be applied to heterogeneous image datasets even when different markers are used. Moreover, we have illustrated how single cell-based measures allow characterisation of tissue topology and aid classification of perturbation effects.

Simplifying the image analysis task to a cell detection problem offers multiple advantages. Firstly, collecting annotations for cell localisation (one cursor click per cell) is much less time-consuming, compared with segmentation (drawing the contour for each cell). Secondly, this approach is valuable in situations when annotation of the cell boundary is not possible, for example, when only nuclear staining is available. Thirdly, segmentation of highly dense cells is a much more challenging task, even when a large number of annotated cells are used [13], [14]. Furthermore, cell detection is less computationally intensive and more suited to high throughput applications that require quantification of thousands of cells per sample. Thus, our approach is more scalable to studies that use many timepoints or live imaging data. The main limitation of our approach is that we cannot obtain accurate measures of cell morphology. However, Voronoi tessellation can still be used to provide approximate measures of cell shapes, areas and densities in confluent monolayers. Therefore, DeepScratch provides a scalable and flexible approach for analysing wound healing imaging data.

The main advantage of using convolutional networks, versus traditional analysis pipelines, is that the former do not require tailored parametrisation of expected cell shapes or marker distribution. For example, DeepScratch performs well under various genetic perturbations that result in substantially different cell morphologies, such as CDH5 knockdown (Fig. 2), assuming that it is trained on a representative subset of images. Thus, this method can be applied to cell lines that have highly heterogenous morphologies, such as neuronal and cancer cells. Furthermore, this approach, along with the proposed metrics, can be applied to a wide range of imaging studies in which object segmentation is a challenging task.

Topological analysis of endothelial cell connectivity revealed that these cells follow similar patterns to epithelial cells from other tissue types [21]. The distribution of polygon shapes in endothelial cells cultured *in vitro* peaks at six sides with a long right tail. Consistent with Lewis’ Law, the number of sides is correlated with cell area. Our study shows that endothelial sheets are composed of, on average, 7% tetragons, 28% pentagons, 36% hexagons, 20% heptagons and 6% octagons. This distribution is strikingly similar to the distribution reported for mutated ﻿*Arabidopsis thaliana* leaves (7% tetragons, 29% pentagons, 36% hexagons, 23% heptagons, 5% octagons) [19], showing that these topologies are highly conserved in nature because they are biophysically constrained. We also observed some extent of spatial correlation; some shapes tended to co-occur with each other more frequently, especially hexagons. Findings of previous studies have varied regarding spatial correlation [19], [24]. We speculate that this correlation is due to cells’ tendency to give rise to certain shapes during cell division [22]. However, more work is needed to identify the biological function underlying cell clustering and its variability in different tissue types.

Our analysis reveals that spatial coordination of cellular spreading is induced upon scratch wounding. We observed that cells closer to the wound are more spread out. However, cells that are more distant from the wound have higher local cell density and are less spread out. This finding might indicate that proliferation rate increases in cells away from the wound, which can be activated by cells at the leading edge [34]. Both CDC42 and CDH5 knockdowns significantly reduce the spatial coordination of cell density, reflecting the importance of such coordination in collective cell migration. However, the depletion of these genes affects different mechanisms; the disruption of cell adhesion through CDH5 knockdown reduces cellular tension, while CDC42 knockdown affects cell response to microenvironmental change [35], [36]. By considering multiple LCD-based measures we can discriminate among the different effects of these perturbations (Fig. 5E). Thus, our interpretable features allow better characterisation of perturbation effects and can provide insights into their biological mechanisms.

In summary, DeepScratch is a useful approach for studying topological changes during collective migration in confluent monolayers. Applying DeepScratch to datasets in which thousands of genetic and pharmacological perturbations are tested will advance our understanding of the roles these factors play in tissue remodelling and cell migration. This can be highly valuable for understanding various disease processes and engineering regenerative medicine approaches.

## Declaration of Competing Interest

The authors declare that they have no known competing financial interests or personal relationships that could have appeared to influence the work reported in this paper.

## Authors Contribution

HS conceived and conceptualised the study. HS and AJ developed and validated the methodology. HS analysed the results and wrote the original manuscript. HS, AJ, and JR reviewed and edited the manuscript.
